# Advancements and Applications of Artificial Intelligence in Pharmaceutical Sciences: A Comprehensive Review

**DOI:** 10.5812/ijpr-150510

**Published:** 2024-10-15

**Authors:** Negar Mottaghi-Dastjerdi, Mohammad Soltany-Rezaee-Rad

**Affiliations:** 1 Department of Pharmacognosy and Pharmaceutical Biotechnology, School of Pharmacy, Iran University of Medical Sciences, Tehran, Iran; 2 Behestan Innovation Factory, Behestan Darou, Tehran, Iran

**Keywords:** Artificial Intelligence, Biotechnology, Clinical Pharmacy, Medicinal Chemistry, Personalized Medicine, Nanotechnology, Pharmaceutical Management, Pharmacognosy, Pharmacology, Toxicology

## Abstract

Artificial intelligence (AI) has revolutionized the pharmaceutical industry, improving drug discovery, development, and personalized patient care. Through machine learning (ML), deep learning, natural language processing (NLP), and robotic automation, AI has enhanced efficiency, accuracy, and innovation in the field. The purpose of this review is to shed light on the practical applications and potential of AI in various pharmaceutical fields. These fields include medicinal chemistry, pharmaceutics, pharmacology and toxicology, clinical pharmacy, pharmaceutical biotechnology, pharmaceutical nanotechnology, pharmacognosy, and pharmaceutical management and economics. By leveraging AI technologies such as ML, deep learning, NLP, and robotic automation, this review delves into the role of AI in enhancing drug discovery, development processes, and personalized patient care. It analyzes AI's impact in specific areas such as drug synthesis planning, formulation development, toxicology predictions, pharmacy automation, and market analysis. Artificial intelligence integration into pharmaceutical sciences has significantly improved medicinal chemistry, drug discovery, and synthesis planning. In pharmaceutics, AI has advanced personalized medicine and formulation development. In pharmacology and toxicology, AI offers predictive capabilities for drug mechanisms and toxic effects. In clinical pharmacy, AI has facilitated automation and enhanced patient care. Additionally, AI has contributed to protein engineering, gene therapy, nanocarrier design, discovery of natural product therapeutics, and pharmaceutical management and economics, including marketing research and clinical trials management. Artificial intelligence has transformed pharmaceuticals, improving efficiency, accuracy, and innovation. This review highlights AI's role in drug development and personalized care, serving as a reference for professionals. The future promises a revolutionized field with AI-driven methodologies.

## 1. Context

Artificial intelligence (AI) has revolutionized various scientific areas, including pharmaceutical sciences (1). This technology, which includes machine learning (ML), deep learning, natural language processing (NLP), and robotic automation, has transformed all fields of pharmaceutical sciences, including research and development as well as clinical practice (2).

Artificial intelligence systems use algorithms to process large datasets, identifying patterns and making predictions through training. By adjusting parameters, these models learn to minimize errors. Once trained, they analyze new data, offering insights and performing tasks such as data analysis, pattern recognition, and predictive modeling with high accuracy and efficiency (3).

**Figure 1.**
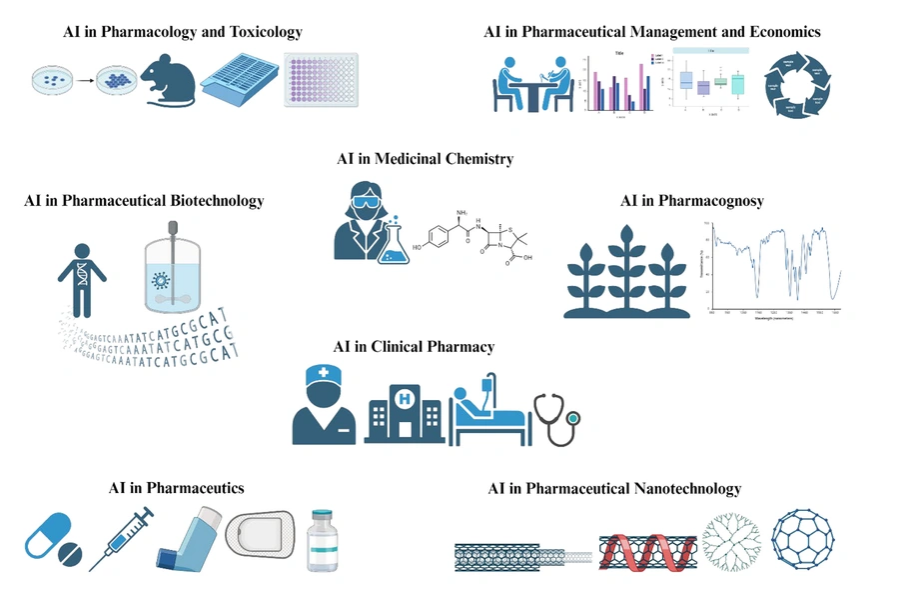
Different fields of pharmaceutical sciences in which artificial intelligence (AI) can be applied (created with BioRender.com).

**Figure 2.**
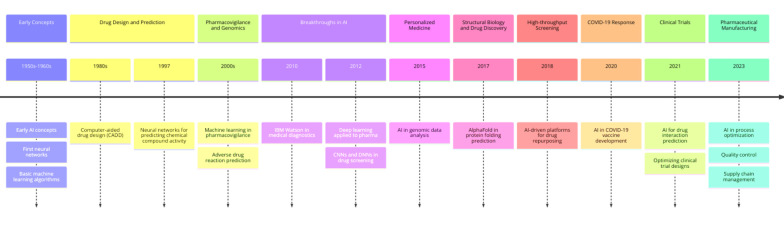
Timeline of the development of artificial intelligence (AI) application in pharmaceutical sciences

There have been numerous challenges in the pharmaceutical industry, including lengthy drug discovery processes, extensive development costs, intricate procedures for drug design, and strict therapeutic intervention requirements (4). Artificial intelligence integration into this field has given pharmaceutical professionals hope to overcome these challenges by improving efficiency, accuracy, and innovation (5). The ability of AI to process large amounts of data, extract patterns, analyze information, and make predictions positions AI as a pivotal aspect of research and development, offering unique opportunities for innovation (6). Figure 1 and Figure 2 illustrate AI's application in pharmaceutical sciences and its historical development. The article provides a comprehensive overview of AI models and their practical applications across various fields, serving as a valuable reference for professionals integrating AI into their work.

## 2. Overview of Artificial Intelligence Models Utilized in Pharmaceutical Sciences

In pharmaceutical sciences, various AI models are employed for different purposes, each offering distinct capabilities and applications. Machine learning models, such as supervised learning, are fundamental in this field. Supervised learning involves training models using labeled datasets to make predictions or decisions. Techniques like regression models, support vector machines (SVM), and decision trees are commonly used. For instance, SVMs classify drug compounds based on chemical properties, aiding drug discovery. Regression models predict drug efficacy and pharmacokinetic properties, while decision trees assist in predicting patient responses and optimizing drug formulations (7).

Unsupervised learning, another crucial type of ML, focuses on uncovering patterns in unlabeled data. Clustering algorithms group similar compounds based on chemical properties, helping identify potential drug candidates. Principal component analysis (PCA) reduces data dimensionality, preserving essential information and revealing patterns that guide drug discovery (8).

Deep learning models, including Convolutional Neural Networks (CNNs) and Recurrent Neural Networks (RNNs) with long short-term memory (LSTM) networks, are pivotal. Convolutional Neural Networks excel in analyzing structured data like images, which is useful in medical imaging for disease diagnosis and drug repurposing. Recurrent Neural Networks and LSTMs process sequential data, predicting patient outcomes and identifying novel drug indications by analyzing relationships between drugs and patient characteristics (9).

Natural language processing is critical in handling unstructured text data, such as scientific literature and electronic health records (EHRs). Natural language processing extracts drug-target interactions and other crucial insights, supporting drug discovery and improving patient care. It also aids in mining medical records for adverse drug reactions (ADR), enhancing pharmacovigilance (10).

Reinforcement learning (RL) involves an agent learning decision-making by interacting with an environment and receiving feedback. In pharmaceutical sciences, RL optimizes clinical trial designs and personalizes treatment plans. It helps in designing adaptive clinical trials, optimizing patient recruitment, and adjusting treatment protocols based on individual patient responses (11).

## 3. Artificial Intelligence in Medicinal Chemistry

**Figure 3.**
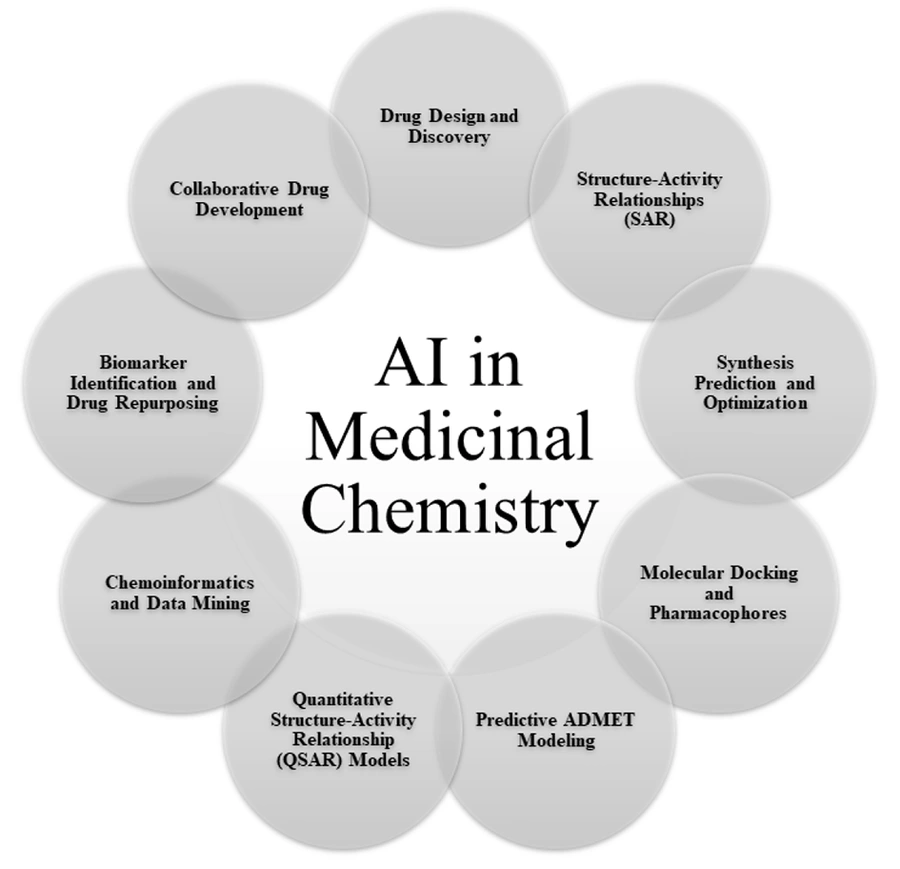
Role of artificial intelligence (AI) in medicinal chemistry

Artificial intelligence's integration into medicinal chemistry marks a significant evolution from traditional pharmaceutical discovery methods, offering new avenues for drug design, synthesis, and optimization (12). The high costs and complexity associated with drug development, particularly due to the high attrition rates in clinical trials and the complexities of the drug discovery phase, necessitate more efficient approaches (5). Artificial intelligence provides these by processing vast amounts of data, predicting molecular behaviors, and simulating drug interactions with greater speed and accuracy. This part will explore deeper into the role of AI in medicinal chemistry (Figure 3) and look at its impact on the field.

### 3.1. Drug Design and Discovery

Artificial Intelligence methodologies, including ML and deep learning, significantly accelerate drug discovery. By analyzing extensive chemical libraries and biological data, AI predicts the efficacy and safety of molecules, thereby enhancing the initial stages of drug discovery (13, 14).

### 3.2. Structure-Activity Relationships

Artificial intelligence models predict how chemical structures relate to biological activities, facilitating the design of molecules with specific therapeutic effects and minimal side effects. These models help identify critical structural features that influence biological activity, thus improving lead compounds (15-18).

### 3.3. Synthesis Prediction and Optimization

Artificial intelligence predicts efficient synthetic pathways, reducing the resources required for drug synthesis. It also predicts reaction outcomes and identifies novel synthesis routes, streamlining the entire drug development process (19).

### 3.4. Molecular Docking and Pharmacophores

Artificial intelligence models interactions between drugs and biological targets, aiding in molecular docking and pharmacophore identification. This assists in discovering new compounds with desired biological activities (20).

### 3.5. Predictive Absorption, Distribution, Metabolism, Excretion, and Toxicity Modeling

Predicting absorption, distribution, metabolism, excretion, and toxicity (ADMET) properties is crucial in drug discovery. Artificial intelligence significantly aids in this area by providing accurate predictions, essential for assessing new compounds' drug-likeness and potential success (21). The detailed discussion on predictive ADMET modeling has been integrated into the "Predictive ADMET and PK/PD Modeling" subsection under "AI in Pharmacology and Toxicology."

### 3.6. Quantitative Structure-Activity Relationship Models

Artificial intelligence-driven quantitative structure-activity relationship (QSAR) models predict the activity of compounds based on their chemical structures, crucial for identifying promising compounds for further analysis (15).

### 3.7. Chemoinformatics

Artificial intelligence extracts valuable insights from large chemoinformatics databases, identifying patterns and relationships between chemical compounds and their biological activities (22).

### 3.8. Biomarker Identification and Drug Repurposing

Artificial intelligence identifies potential disease-associated biomarkers and discovers new therapeutic applications for existing drugs, facilitating drug repurposing efforts (23). The detailed discussion on biomarker identification and drug repurposing has been integrated into the "Biomarker Discovery, Validation, and Drug Repurposing" subsection under "AI in Pharmaceutical Biotechnology."

### 3.9. Collaborative Drug Development

Artificial intelligence enhances collaboration among chemists, biologists, and data scientists, expediting the discovery of new medicines (24, 25).

### 3.10. Relevant Studies

Several studies highlight AI's impact in medicinal chemistry. For example, Harren et al. utilized explainable artificial intelligence (XAI) methods in lead optimization, providing valuable SAR insights. Matsuzaka et al.'s (as cited by Harren et al.) DeepSnap-DL system improved QSAR predictions (18). Other studies have demonstrated AI's role in synthesis planning, predictive ADMET modeling, and drug repurposing, underscoring the technology's transformative potential (Appendix 1 in Supplementary File).

## 4. Artificial Intelligence in Pharmaceutics

**Figure 4.**
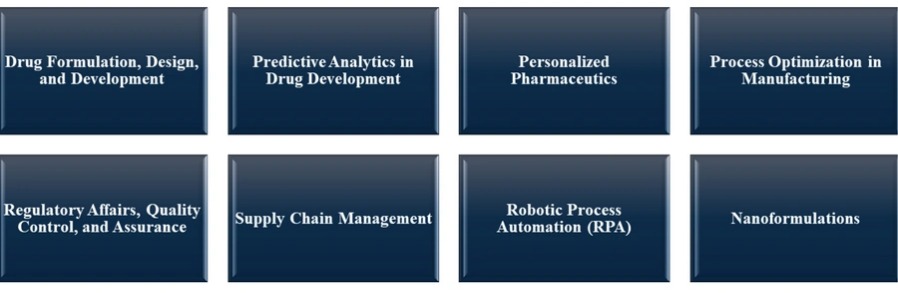
Impact of artificial intelligence (AI) in the field of pharmaceutics

Artificial intelligence is profoundly transforming the field of pharmaceutics by enhancing the development, formulation, and manufacturing of pharmaceutical products. Its capabilities in data analysis, predictive modeling, and automation are driving efficiency, accuracy, and innovation across the industry. In this section, we will discuss how AI is impacting the field of pharmaceutics (Figure 4). 

### 4.1. Drug Formulation, Design, and Development

Artificial intelligence algorithms evaluate data to predict the stability and compatibility of pharmaceutical ingredients. This technology can improve formulations for controlled release, optimize bioavailability, and minimize side effects, enhancing the entire lifecycle of pharmaceutical products (25-27).

### 4.2. Predictive Analytics in Drug Development

Artificial intelligence models are used to predict how drug formulations will behave under various conditions, aiding in the assessment of efficacy and stability. This is particularly crucial for understanding the physicochemical properties of drugs, such as solubility and permeability, which are critical factors in pharmacokinetics (PK) and target receptor interactions (28).

### 4.3. Personalized Pharmaceutics

Artificial intelligence enables the development of personalized drug formulations tailored to individual patient needs, considering genetic, lifestyle, and medical factors. The push towards personalized medicine is exemplified by initiatives like the precision medicine initiative (PMI) (29). Artificial intelligence also addresses the challenge of providing flexible dosage strengths, crucial for drugs with a narrow therapeutic index, through innovations like data-enriched edible pharmaceuticals (DEEP) (30).

### 4.4. Process Optimization in Manufacturing

Artificial intelligence tools improve manufacturing processes by predicting ideal conditions, monitoring quality, and forecasting potential issues, thus enhancing efficiency and reducing costs (31, 32).

### 4.5. Regulatory Affairs, Quality Control, and Assurance

Artificial intelligence tools streamline regulatory processes and improve quality control in pharmaceutical manufacturing. They automate tasks such as auditing, quality management, dossier filling, and compliance monitoring, ensuring that products meet regulatory standards and identifying impurities quickly (33, 34).

### 4.6. Supply Chain Management

The role of AI in demand forecasting, inventory management, and distribution optimization is crucial for ensuring timely delivery of pharmaceutical products while avoiding shortages or overstocks (35). The detailed discussion on supply chain management has been integrated into the "Supply Chain Optimization" subsection under "AI in Pharmaceutical Management and Economics."

### 4.7. Robotic Process Automation

In pharmaceutical manufacturing, robotic process automation (RPA) is employed for repetitive tasks such as packaging and labeling to enhance efficiency and minimize human error (36).

### 4.8. Nanoformulations

Pharmaceutical nanotechnology utilizes AI to create and refine nano-scale drug delivery systems, which can precisely target specific areas of the body, resulting in more effective treatments with fewer side effects (37).

### 4.9. Relevant Studies

Examples of AI applications in pharmaceutics include the Formulation AI platform for in silico formulation design, which predicts critical properties of drug formulations, and RPA systems that automate business processes. These technologies demonstrate AI's capability to streamline drug product development and optimize manufacturing processes (38). For detail refer to Appendix 1 in Supplementary File.

## 5. Artificial Intelligence in Pharmacology and Toxicology

**Figure 5.**
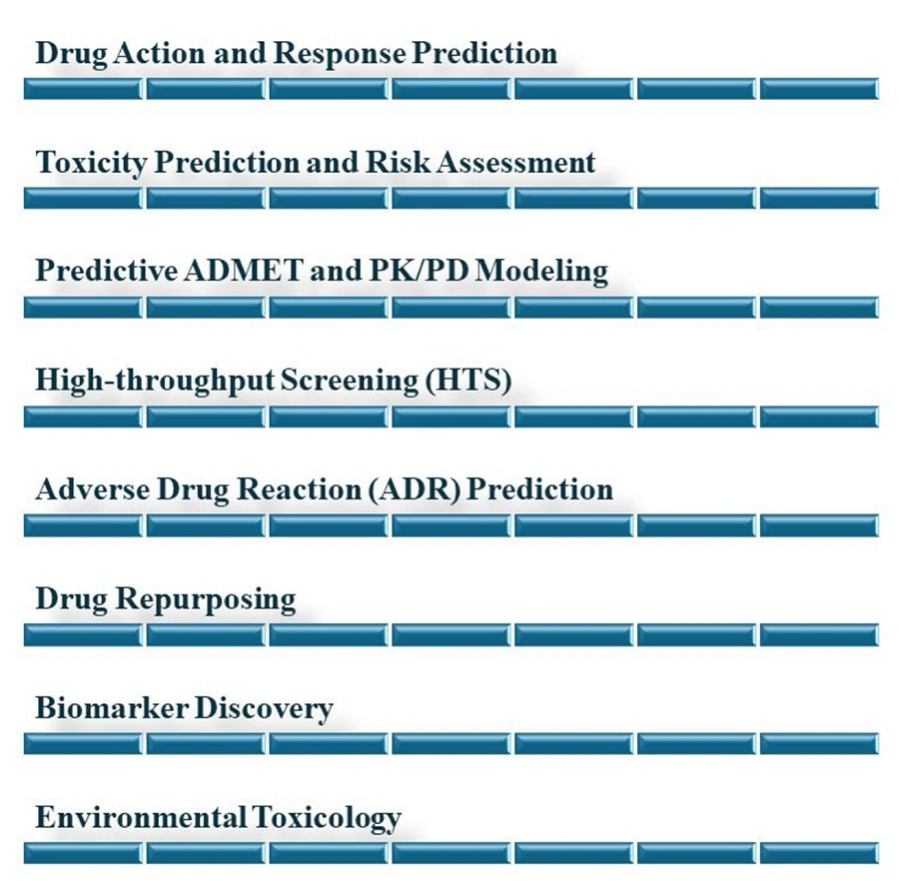
Artificial intelligence (AI) in pharmacology and toxicology

The integration of AI into pharmacology and toxicology marks a significant advancement, offering profound insights into drug effects, safety profiles, and environmental impacts. Artificial intelligence's ability to manage large datasets, identify patterns, and predict outcomes is revolutionizing these fields, providing a comprehensive understanding of pharmacological research and toxicological risk assessment. This part of the article elucidates the contribution of AI in these fields (Figure 5). 

### 5.1. Drug Action and Response Prediction

Artificial intelligence algorithms analyze complex datasets to predict the pharmacological effects and mechanisms of action of drugs. This capability allows for the development of more targeted and effective treatments by anticipating drug interactions within biological systems (39, 40).

### 5.2. Toxicity Prediction and Risk Assessment

In toxicology, AI plays a pivotal role by predicting the potential toxicity of substances. By analyzing chemical structures and historical toxicity data, ML algorithms can forecast the toxicity of new compounds, reducing reliance on extensive in vivo testing (41, 42).

### 5.3. Predictive Absorption, Distribution, Metabolism, Excretion, and Toxicity and Pharmacokinetics /Pharmacodynamics Modeling

Artificial intelligence significantly contributes to predicting and modeling ADMET properties, crucial in drug discovery. Additionally, AI models simulate PK and pharmacodynamics (PD), helping to understand drug behavior in the body and optimize dosing, ensuring efficacy and safety (43, 44).

### 5.4. High-Throughput Screening

Artificial intelligence accelerates high-throughput screening (HTS) by quickly analyzing vast amounts of pharmacological test data, facilitating the rapid identification of potential compounds and enhancing the understanding of their pharmacological properties (45). The detailed discussion on HTS has been integrated into the "High-throughput Screening" subsection under "AI in Pharmaceutical Biotechnology."

### 5.5. Adverse Drug Reaction Prediction

Artificial intelligence can analyze medical records and patient feedback to predict ADR s, helping to ensure drug safety by detecting rare but severe side effects (46).

### 5.6. Drug Repurposing

By examining existing drugs' characteristics and effects on various biological targets, AI identifies new therapeutic uses for them. This cost-effective method shortens the development time compared to creating entirely new drugs (47). The comprehensive discussion on Drug Repurposing has been integrated into the "AI in Pharmaceutical Biotechnology" section, specifically under the "Biomarker Discovery, Validation, and Drug Repurposing" subsection.

### 5.7. Biomarker Discovery

Artificial intelligence is instrumental in identifying biomarkers for drug development, diagnosis, and treatment monitoring. This capability fosters the creation of targeted therapies and personalized medicine approaches (48). A detailed discussion is provided in the "Biomarker Discovery, Validation, and Drug Repurposing" subsection in the "AI in Pharmaceutical Biotechnology" section.

### 5.8. Environmental Toxicology

Artificial intelligence models assess the impact of chemicals on the environment and public health, aiding in the development of safer chemicals and environmental guidelines (49).

### 5.9. Relevant Studies

Recent research highlights AI's transformative role in pharmacology and toxicology. For instance, studies have explored the prognostic and therapeutic significance of various genes in gastric cancer and the regulatory role of miRNAs in glioblastoma. Machine learning models have predicted clinical responses to anti-epileptic drugs and ADRs, demonstrating the integration of clinical and genetic data. Advances in chemical language models for toxicity prediction and the use of AI in ADMET modeling underscore AI's growing importance in preclinical drug discovery (50, 51). More relevant studies are provided in Appendix 1 in Supplementary File.

## 6. Artificial Intelligence in Clinical Pharmacy

**Figure 6.**
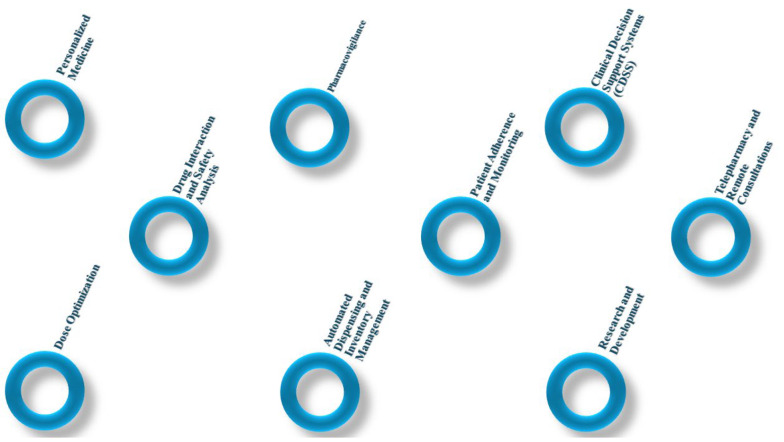
How artificial intelligence (AI) is being integrated into clinical pharmacy

Artificial intelligence is increasingly being used in clinical pharmacy to aid in providing better patient care. By utilizing AI's capabilities in data analysis, pattern recognition, and forecasting, clinical pharmacy can implement more accurate, efficient, and customized methods. Artificial intelligence can enhance various aspects of clinical pharmacy, including drug dispensing and patient counseling, and this section provides a detailed analysis of how AI is being integrated into clinical pharmacy (Figure 6). 

### 6.1. Personalized Medicine

Artificial intelligence algorithms are used to analyze patient data, including genetic information, to customize drug therapies. This personalized approach ensures that patients are prescribed medications that are most effective for their unique profiles, leading to better treatment outcomes and fewer adverse reactions (52).

### 6.2. Drug Interaction and Safety Analysis

Artificial intelligence systems are capable of rapidly analyzing a patient's medication list to identify possible drug-drug interactions and contraindications. This process helps to lower the chance of ADRs and improve patient safety (39).

### 6.3. Dose Optimization

Artificial intelligence aids in determining the most suitable dosage of medication for individual patients. It considers various factors such as age, weight, kidney function, and specific patient conditions to suggest doses that optimize efficacy while minimizing side effects (53).

### 6.4. Pharmacovigilance

Artificial intelligence tools monitor and analyze data from different sources to identify possible ADRs and medication errors, providing a proactive approach to pharmacovigilance. This process helps to enhance patient safety and improve drug profiles (54).

### 6.5. Patient Adherence and Monitoring

Applications and devices powered by AI can track patient adherence to medication regimens, send reminders, and notify healthcare providers if patients are not following prescribed therapies (55).

### 6.6. Automated Dispensing and Inventory Management

Artificial intelligence enhances the efficiency of pharmacy operations by automating dispensing processes and managing inventory. This not only saves time but also reduces human error in dispensing medications (56, 57).

### 6.7. Clinical Decision Support Systems

Artificial intelligence-powered clinical decision support systems (CDSS) provide pharmacists and healthcare providers with evidence-based guidance and recommendations, assisting in clinical decision-making and ensuring the best patient care (58).

### 6.8. Telepharmacy and Remote Consultations

Artificial intelligence facilitates advanced telepharmacy services by enabling patients to receive consultations and medication management advice remotely, increasing accessibility to pharmaceutical care (59).

### 6.9. Research and Development

Artificial intelligence is utilized in clinical pharmacy research to scrutinize clinical trial data, patient outcomes, and treatment efficacies, advancing better therapeutic protocols and drug policies (60, 61).

### 6.10. Relevant Studies

Yin et al. reviewed 51 studies from 2010 to 2020, highlighting AI's use in clinical settings for screening, diagnosis, risk analysis, and treatment of various conditions (62). The xDECIDE system exemplifies AI's role in personalized oncology care by integrating real-world evidence and expert knowledge (63). Other studies demonstrate AI's potential in personalized medicine and drug repurposing, such as the DRIAD framework for Alzheimer's disease. More studies can be found in Appendix 1 in Supplementary File.

## 7. Artificial Intelligence in Pharmaceutical Biotechnology

**Figure 7.**
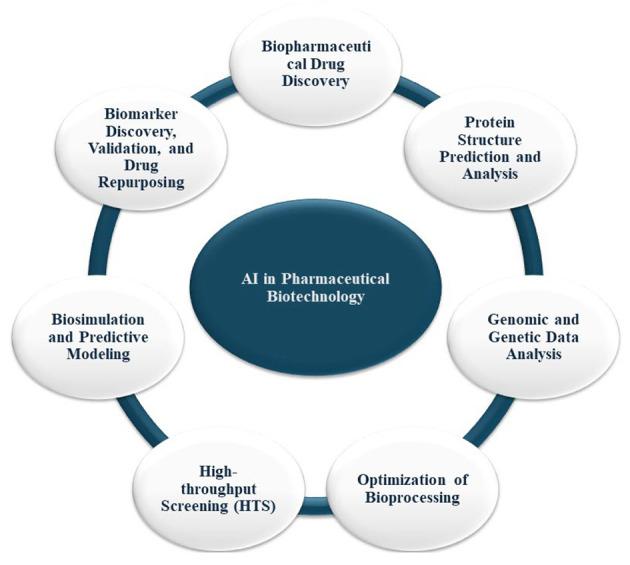
Artificial intelligence (AI)'s use in pharmaceutical biotechnology

Artificial intelligence is increasingly integral to pharmaceutical biotechnology, revolutionizing research methodologies and expediting the development of biopharmaceutical products. Its capabilities in data analysis, predictive modeling, and process optimization are particularly valuable for navigating the complexities of biological systems and biotechnological processes. This section outlines AI's use in pharmaceutical biotechnology (Figure 7). 

### 7.1. Biopharmaceutical Drug Discovery

Artificial intelligence algorithms identify potential biopharmaceutical drugs such as antibodies, vaccines, and gene therapies. By analyzing biological data, AI can predict the efficacy and safety of these complex molecules, significantly accelerating the discovery process (64-66). For example, a systems biology approach has provided insights into gastric cancer pathogenesis, revealing complex gene networks and pathways (67).

### 7.2. Protein Structure Prediction and Analysis

Artificial intelligence, especially deep learning models, predicts the 3D structures of proteins, essential for understanding protein functions and designing drugs that target these proteins accurately (68).

### 7.3. Genomic and Genetic Data Analysis

Artificial intelligence analyzes vast genomic data to identify genetic markers associated with diseases and potential therapeutic targets, aiding in the development of personalized medicine strategies and targeted therapies (69).

### 7.4. Optimization of Bioprocessing

Artificial intelligence optimizes biotechnological processes, including fermentation, cell culture, and purification. Machine Learning models predict optimal conditions, improving efficiency and yield (70).

### 7.5. High-Throughput Screening

Artificial intelligence plays a crucial role in accelerating HTS in both drug discovery and biopharmaceutical research. By quickly analyzing the outcomes of thousands of pharmacological tests, AI speeds up the identification of potential compounds and enhances the understanding of their pharmacological properties. Additionally, AI's ability to rapidly analyze data from a wide range of experiments helps identify potential therapeutic targets and biological compounds more efficiently. This integration of AI in HTS makes the process more streamlined and effective, significantly contributing to advancements in both pharmacology and biopharmaceutical development (45).

### 7.6. Biosimulation and Predictive Modeling

Artificial intelligence-powered biosimulation models mimic biological processes and drug interactions, providing insights into drug interactions, side effects, and potential therapeutic pathways (71).

### 7.7. Biomarker Discovery, Validation, and Drug Repurposing

Artificial intelligence plays a crucial role in enhancing both the discovery and validation of biomarkers, as well as facilitating drug repurposing efforts. By analyzing large datasets, AI can efficiently and accurately identify potential disease-associated biomarkers, essential for diagnosing diseases and developing targeted therapies. Furthermore, AI can analyze the characteristics and effects of existing drugs on various biological targets to identify novel therapeutic applications. This approach to drug repurposing is not only cost-efficient but also significantly reduces the development timeline compared to creating entirely new drugs. Utilizing AI in this manner accelerates the drug development process and enhances the potential for finding effective treatments for various conditions (72, 73).

### 7.8. Relevant Studies

Noteworthy studies include Sumitomo Dainippon Pharma's collaboration with Exscientia, which rapidly discovered DSP-1181 for obsessive-compulsive disorder. Artificial intelligence algorithms have also been used to predict the immunogenicity of biologic candidates, improving clinical translation success rates (74). Furthermore, AI is advancing cell and gene therapies by identifying optimal targets and designing delivery vehicles. In COVID-19 research, AI-based feature selection methods have provided insights into potential biomarkers (75). Additionally, AI's role in drug repurposing and biopharmaceutical innovation is expanding globally, with numerous companies exploring its applications (76). More studies are provided in Appendix 1 in Supplementary File.

## 8. Artificial Intelligence in Pharmaceutical Nanotechnology

**Figure 8.**
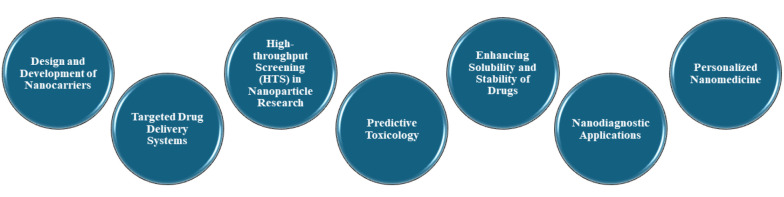
Role of artificial intelligence (AI) in pharmaceutical nanotechnology

Artificial intelligence is significantly advancing pharmaceutical nanotechnology, particularly in the development and application of nanomedicines. Its capabilities in data analysis, predictive modeling, and design optimization are ideally suited for the complex requirements of nanotechnology. This section will explore the role of AI in this field (Figure 8). 

### 8.1. Design and Development of Nanocarriers

Artificial intelligence algorithms are increasingly used to design nanocarriers for drug delivery, predicting optimal dimensions, shapes, and surface properties to improve targeting accuracy and reduce toxicity, thereby enhancing therapeutic effectiveness (77).

### 8.2. Targeted Drug Delivery Systems

Artificial intelligence aids in creating targeted drug delivery systems, predicting interactions between nanoparticles and biological systems to enhance precision in drug targeting, thereby minimizing side effects and improving efficacy (37).

### 8.3. High-Throughput Screening in Nanoparticle Research

Artificial intelligence facilitates the rapid analysis of various nanoparticle formulations for drug delivery and imaging, streamlining the HTS process in nanoparticle research (78).

### 8.4. Predictive Toxicology

Artificial intelligence models are critical for predicting the potential toxicity of nanoparticles, analyzing physicochemical properties to assess biocompatibility and environmental impact, ensuring the safety of nanomedicines (79).

### 8.5. Enhancing Solubility and Stability of Drugs

Artificial intelligence assists in improving the solubility and stability of poorly soluble drugs through nanoformulation, predicting ideal nanocarriers and formulation techniques to enhance drug solubility (77).

### 8.6. Nanodiagnostic Applications

Artificial intelligence contributes to the development of nanotechnology-based sensors and diagnostic devices, including nanoscale biosensors and diagnostic assays, for the early and accurate detection of diseases (80, 81).

### 8.7. Personalized Nanomedicine

With AI, pharmaceutical nanotechnology is moving towards personalized medicine, using patient-specific data to tailor nanomedicine formulations, thereby maximizing therapeutic outcomes (82).

### 8.8. Process Optimization in Nanoparticle Manufacturing

Artificial intelligence is used to optimize nanoparticle manufacturing processes, improving scalability, consistency, and quality control in production (83).

### 8.9. Relevant Studies

In collaboration with AstraZeneca, researchers at Cardiff University designed AI-driven nanoparticles for delivering mRNA to cancer cells, demonstrating superior efficacy compared to other prototypes (84, 85). A study by Banaye Yazdipour et al. (86) employed AI tools like Random Forest and Support Vector Machine to predict the toxicity of metal oxide and metallic nanoparticles, offering a fast and cost-effective alternative to traditional toxicity testing methods. Numerous studies highlight the potential of AI in accelerating drug development and discovering new treatments, with interdisciplinary collaborations among pharmaceutical scientists, computer scientists, statisticians, and physicians playing a crucial role (24, 25). More relevant studies are provided in Appendix 1 in Supplementary File.

## 9. Artificial Intelligence in Pharmacognosy

**Figure 9.**
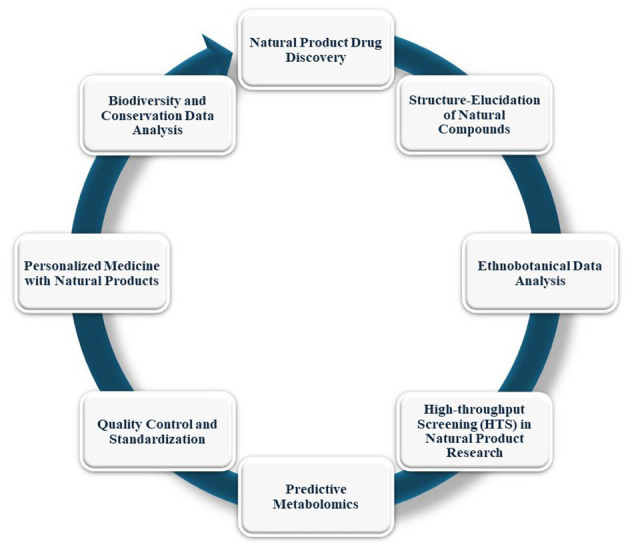
The impact of artificial intelligence (AI) on pharmacognosy

The identification and examination of natural chemicals are changing because of the incorporation of AI into pharmacognosy, the study of medications obtained from natural sources. This discipline benefits greatly from AI's ability to handle big datasets, anticipate compound qualities, and clarify intricate biological connections. This section discusses the impact of AI on pharmacognosy (Figure 9). 

### 9.1. Natural Product Drug Discovery

Artificial intelligence expedites the identification and analysis of bioactive compounds from natural sources such as marine organisms, fungi, and plants. Machine Learning algorithms can swiftly screen large natural product libraries, identifying potential therapeutic compounds more efficiently than traditional methods (87).

### 9.2. Structure-Elucidation of Natural Compounds

Using spectral data, Artificial Intelligence can anticipate the structure of new natural substances. Understanding the bioactivity of these molecules and accelerating their development into medications depend on this quick structural elucidation (88).

### 9.3. Ethnobotanical Data Analysis

Artificial intelligence algorithms are increasingly used to analyze ethnobotanical data, merging traditional medicinal knowledge with modern drug discovery. This involves identifying potential drug leads by studying the historical and cultural uses of natural substances (89).

### 9.4. High-Throughput Screening in Natural Product Research

In pharmacognosy, AI improves the effectiveness of HTS techniques by enabling the quick screening of multiple natural extracts and compounds against different biological targets (90).

### 9.5. Predictive Metabolomics

Artificial intelligence plays a significant role in metabolomics research by predicting the metabolic pathways of natural products and elucidating their mechanisms of action, which is essential for assessing their medicinal potential (91).

### 9.6. Quality Control and Standardization

Artificial intelligence applications are vital for the quality control and standardization of herbal medicines. They analyze chemical profiles and detect contaminants, ensuring the safety and reliability of natural products (92).

### 9.7. Personalized Medicine with Natural Products

Scientists are creating AI models to customize natural product-based treatments for individual patients. This involves assessing patient-specific information to anticipate the reactions to herbal medicines (52, 93).

### 9.8. Biodiversity and Conservation Data Analysis

Artificial intelligence helps in scrutinizing biodiversity data to recognize and preserve medicinal plants and other organisms that are reservoirs of precious natural compounds (94).

### 9.9. Relevant Studies

Desai et al. highlighted AI's role in enhancing pharmacognostic performance, particularly in identifying active compounds and optimizing extraction processes (95). Gallicchio et al. developed MproPred, a web application utilizing ML to predict compound bioactivity against SARS-CoV-2's main protease, showing significant potential for COVID-19 drug development (96). More relevant studies can be found in Appendix 1 in Supplementary File.

## 10. Artificial Intelligence in Pharmaceutical Management and Economics

**Figure 10.**
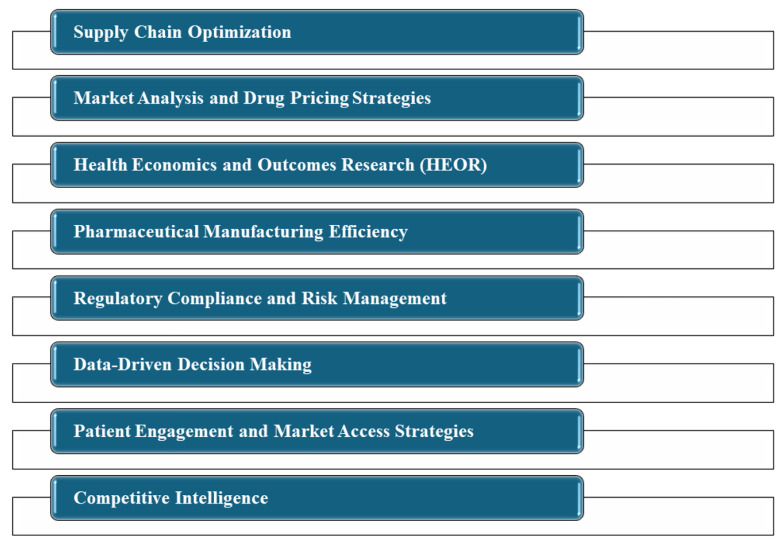
Artificial intelligence (AI) in pharmaceutical management and economics

Artificial intelligence is playing a transformative role in pharmaceutical management and economics, offering solutions to enhance efficiency, decision-making, and economic outcomes. Its capabilities in analyzing complex datasets, predicting market trends, and optimizing operational processes are particularly beneficial in addressing the unique challenges faced by the pharmaceutical industry. In this part, an exploration of AI’s role in this area is provided (Figure 10). 

### 10.1. Supply Chain Optimization

Artificial intelligence improves the management of pharmaceutical supply chains by predicting demand, controlling inventory, and managing distribution logistics. This leads to reduced waste, timely product availability, and cost reduction, as demonstrated by studies showcasing optimized inventory levels and streamlined distribution processes (97).

### 10.2. Market Analysis and Drug Pricing Strategies

Artificial intelligence is crucial in analyzing pharmaceutical market trends and consumer behavior, helping companies make informed decisions on drug marketing, pricing, and sales. It predicts the market potential of new drugs and assesses the impact of pricing strategies on market share and profitability (98).

### 10.3. Health Economics and Outcomes Research

Artificial intelligence models analyze large datasets in health economics and outcomes research (HEOR), evaluating the value of pharmaceutical products based on clinical outcomes, quality of life, and cost-effectiveness. This analysis informs decisions on drug development priorities and healthcare policies (99).

### 10.4. Pharmaceutical Manufacturing Efficiency

Artificial intelligence enhances manufacturing efficiency by monitoring production lines, predicting equipment malfunctions (predictive maintenance), and optimizing production schedules. This results in reduced downtime, cost savings, and improved operational efficiency (100).

### 10.5. Regulatory Compliance and Risk Management

Artificial intelligence tools ensure compliance with regulatory standards through automated monitoring and reporting. They also assist in risk management by identifying potential regulatory issues early in the drug development process (100).

### 10.6. Data-Driven Decision Making

Artificial intelligence facilitates strategic decision-making in pharmaceutical management by analyzing clinical trial data, patient outcomes, and market research, providing valuable insights that guide more informed and effective decisions (100).

### 10.7. Patient Engagement and Market Access Strategies

Artificial intelligence -powered platforms enhance patient engagement and support market access strategies by personalizing patient communication and optimizing patient support programs, leading to better patient outcomes and stronger engagement (100).

### 10.8. Competitive Intelligence

Artificial intelligence gathers and analyzes competitive intelligence, allowing pharmaceutical companies to stay ahead in a competitive market by understanding competitors' strategies, market positions, and product developments (5).

### 10.9. Relevant Studies

Studies highlight AI's role in improving manufacturing processes, such as predicting ideal production conditions and monitoring drug quality (31, 32). Artificial intelligence's application in supply chain optimization has led to significant efficiency and cost improvements, as seen in studies focusing on the US pharmaceutical supply chain post-Hurricane Maria (101). More studies are provided in Appendix 1 in Supplementary File.

## 11. Challenges, Limitations, Potential Drawbacks, and Ethical Considerations of Artificial Intelligence

The integration of AI into pharmaceutical sciences offers significant opportunities alongside notable challenges and ethical considerations. Among the primary issues are data privacy and confidentiality, as AI often requires handling sensitive patient data. Safeguarding this information against breaches and unauthorized access, while complying with regulations like GDPR and HIPAA, is crucial (100). Bias and fairness in AI algorithms are also major concerns; AI systems can perpetuate existing disparities if trained on biased data, leading to unequal treatment in drug development and patient care (102).

Transparency and explainability are critical, especially for deep learning models often regarded as 'black boxes' due to their complexity. This lack of transparency can hinder trust and accountability, particularly in healthcare, where understanding the basis for AI-driven decisions is vital (103). Regulatory compliance is another challenge, as AI technologies must adhere to stringent pharmaceutical regulations and standards, requiring validation for clinical use and navigation of complex regulatory pathways (104).

Ethical issues also arise, particularly regarding data privacy, informed consent, and the potential misuse of AI. Clear guidelines and consent processes are necessary to ensure ethical AI deployment (105). The dependency on AI could also lead to a skill gap in the pharmaceutical workforce, necessitating ongoing education and training to keep professionals adept in collaborating with AI technologies (106, 107).

Intellectual property rights pose another challenge, as determining ownership of AI-generated discoveries can be complex (107). The quality and reliability of data are crucial for AI accuracy, yet gathering and maintaining high-quality datasets is challenging in pharmaceutical sciences (108). Additionally, the high cost of integrating AI solutions may limit accessibility, particularly in low-resource settings, potentially widening global healthcare disparities (109).

Overall, while AI promises to revolutionize pharmaceutical sciences, it is essential to address these challenges and ethical considerations to ensure that its benefits are realized responsibly and equitably. The focus should be on leveraging AI's potential in a manner that is fair and beneficial for all stakeholders, avoiding exacerbation of existing inequalities and ensuring robust, ethical AI deployment in healthcare.

## 12. Conclusion and Future Perspectives

**Figure 11.**
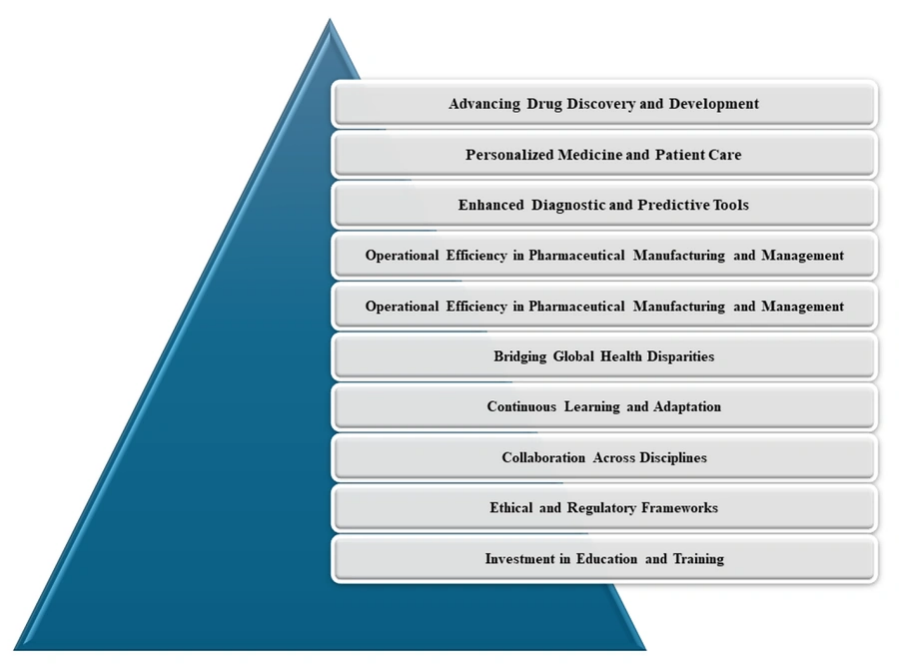
Future perspectives of using artificial intelligence (AI) in the pharmaceutical field

The future integration of AI in pharmaceutical fields (Figure 11) promises significant innovation, efficiency, and personalized medicine. Artificial intelligence's capabilities in analyzing large datasets and simulating biological processes can make drug discovery and development more efficient and cost-effective, potentially halving timelines and reducing costs by up to 40% by 2030 (110). This technology is also expected to enable highly personalized treatment plans, optimize therapeutic outcomes, and minimize side effects, with widespread adoption anticipated in the next decade (111).

Artificial intelligence's role in early disease detection and forecasting treatment responses will revolutionize diagnostic laboratories, increasing precision and speed (112). It will also streamline manufacturing and supply chain processes, potentially boosting operational efficiency by 30%. Furthermore, AI can democratize access to advanced healthcare, reducing disparities between high-resource and low-resource settings (113).

As AI systems evolve, they will continuously learn and adapt, ensuring ongoing innovation in pharmaceutical practices. By 2035, AI is expected to be seamlessly integrated into global healthcare systems, necessitating robust ethical and regulatory frameworks to ensure patient safety, data privacy, and equitable access to AI-driven therapies. Increased collaboration among AI experts, pharmaceutical scientists, healthcare providers, and policymakers will be crucial (6, 113).

Investment in education and training is essential to equip professionals with the skills to work alongside AI technologies (114). While the potential of AI in pharmaceuticals is vast, it is crucial to address ethical, regulatory, and practical challenges to ensure responsible and equitable application of these technologies, ultimately improving global healthcare (115).

ijpr-150510-Supplementary file.pdf

## Data Availability

The data presented in this study are uploaded as a supplementary file during submission and are openly available to readers upon request.
