# Supplementary material for: Advancements and Applications of Artificial Intelligence in Pharmaceutical Sciences: A Comprehensive Review
Source: Iran J Pharm Res. 2024 Oct 15;23(1):e150510. doi: 10.5812/ijpr-150510 (PMC11787549; doi:10.5812/ijpr-150510)
Supplement: ijpr-150510-Supplementary file.pdf [file ijpr-23-1-150510-s001.pdf]

Appendix 1

3. AI in Medicinal Chemistry

3.1. Relevant Studies

Harren *et al.* applied and compared multiple Explainable Artificial Intelligence (XAI) methods to lead optimization data sets with well-established SARs and available X-ray crystal structures. Their results showed that combining DNN models with SHAP-based methods yields easily understandable interpretations, providing valuable insights into the underlying SAR [1]. Matsuzaka *et al.* developed an enhanced DeepSnap-DL system that incorporates the procedures for converting a 3D chemical structure into an image, using deep learning with the image as input data, and statistically evaluating prediction performance. Their improved DeepSnap-DL system is poised to serve as a robust tool for computer-aided molecular design as an innovative quantitative structure-activity relationship (QSAR) system [2]. Griffin *et al.* talked about the general synthetic drug substance process development tasks and highlighted the areas in which machine learning and AI might be beneficially developed and applied [3]. Thakkar *et al.* showed the application of AI in computer-assisted synthesis planning (CASP) in the process of drug discovery and development. They highlighted the necessity for an automated synthesis system that could be used by the chemists to expand their workflows [4]. Johansson *et al.* have provided a comprehensive overview of the recent progress in retro-synthesis planning, forward synthesis prediction, and quantum chemistry-based reaction prediction models. They also discussed the progress from rule-based platforms to AI-assisted methods [5]. Wang *et al.* proposed a deep learning-based computational model called DeepSA, for the prediction of the compounds' synthesis accessibility. This model introduces a helpful tool for the selection of the molecules [6]. Lee *et al.* proposed an AI system supporting the creation of ideal synthetic pathways, especially in the primary research steps and design process. This platform uses a hybrid approach that combines generative evaluation and use of reaction knowledge graphs with machine learning-based retrosynthetic prediction [7]. A recent group of scientists conducted a series of comparative studies between diverse

ligand-based virtual screening methods including deep neural networks and random forest, and showed that DNN and RF outperformed other methods in terms of hit prediction efficiency [8]. A recent review discussed the integration of wet experiments, molecular dynamics simulation, and machine learning techniques to improve QSAR models draw attention to the multidisciplinary method in improving QSAR modeling [9]. Current state of the art progress in the drug development process has been discussed by a group of scientists that focus on analyzing several AI and ML algorithms that have been employed in recent years, with a particular focus on QSAR [10]. Niazi and Mariam provide a comprehensive guide for scientists that helps them know the synergy between chemoinformatics, QSAR, and machine learning [11].

4. AI in Pharmaceutics

4.1. Relevant Studies

Dong *et al.* created FormulationAI, a web-based platform for in silico formulation design. It collects extensive datasets on six widely used drug formulation systems in the pharmaceutical industry over the past decade. The platform intelligently predicts and evaluates 16 important properties, allowing for formulation design by inputting basic information of drugs and excipients. FormulationAI is the first freely available comprehensive web-based platform of its kind, providing a powerful solution to aid formulation design in the pharmaceutical industry. Access it at https://formulationai.computpharm.org/ [12]. Reddy *et al.* illustrated how Robotic RPA integrates AI to automate business processes traditionally performed by humans. They showed the advantages of RPA, such as scalability, reliability, time-saving, and cost-effectiveness, while also addressing challenges such as monetary expense and the need for technical skills [13]. Goud *et al.* explained how AI algorithms are exceptionally well-suited to predict effective drug formulations from various datasets and simplify the drug product development process by minimizing its complications [14].

5. AI in Pharmacology and Toxicology

5.1. Relevant Studies

Investigations into CTGF, FN1, IL-6, THBS1, and WISP1 genes, along with the PI3K-Akt signaling pathway, have identified their prognostic and therapeutic significance in gastric cancer, offering new avenues for targeted therapy [15]. In addition, a comprehensive analysis has proposed a regulatory role for miRNAs on the Wnt/β-catenin signaling pathway and its crosstalk with other cellular pathways in the tumorigenesis of glioblastoma, offering insights into potential therapeutic targets [16].In a study focusing on the anti-epileptic drug brivaracetam, a combination of feature extraction and machine learning was used to integrate clinical and genetic data from a dataset of 235 patients. The resulting model successfully predicted clinical drug response, demonstrating the potential of integrating genetics data with clinical data for prediction. This approach could have a significant impact on reducing clinical study sizes and represents a blueprint for precision medicine in fields such as neurology [17]. Born *et al. in 2023* developed a new interpretable chemical language model that outperforms existing approaches in predicting various molecular properties, including toxicity. The model utilizes SMILES representations with augmentation and introduces two simple methods for uncertainty estimation, leading to improved accuracy. Validation on a large-scale proprietary toxicity dataset demonstrates superior performance in revealing cytotoxic substructures [18]. Liu *et al.* used a machine-learning approach to predict adverse drug reactions (ADRs) by combining drug phenotypic characteristics with chemical and biological properties. They found that phenotypic data was the most informative for ADR prediction, and combining biological and phenotypic features significantly improved the ADR prediction model. This model successfully identified ADRs associated with the withdrawal of rofecoxib and cerivastatin, highlighting the value of phenotypic information for ADR prediction [19]. A recent review discussed how AI is revolutionizing preclinical drug discovery by providing an overview of current AI technologies and highlighting examples where AI has made a real impact [20]. Another review explored new progress made in ADMET modeling in drug discovery, particularly in the context of AI and *in silico* models. Even with the pharmaceutical industry's

problems with late-stage toxicity failures of lead molecules, the review showed significant progress achieved over the last forty years in this field [21].

6. AI in Clinical Pharmacy

6.1. Relevant Studies

Yin *et al.* in 2021 found 51 studies from 2010 to 2020 that used AI in clinical settings. The studies focused on tasks like screening, diagnosis, risk analysis, and treatment for conditions such as sepsis, breast cancer, diabetic retinopathy, and polyps. They assessed the performance and impact of AI applications on clinician and patient outcomes, and in one case, their economic impact. According to this study, the use of AI applications in clinical settings is still in its early stages despite its promising potential [22]. xDECIDE is a clinical decision support system for oncology healthcare providers. It uses a "Human-AI Team" approach to process electronic medical records and create structured health records and standardized clinico-genomic features. Employing an ensemble of AI models called xCORE, the system generates a ranked list of treatment options based on clinical evidence and real-world outcomes, which are then reviewed by a virtual tumor board of experts. The xDECIDE system aims to improve patient outcomes by integrating real-world evidence and expert knowledge into personalized oncology care [23]. Parekh, Ad-Duhaa *et al.* delve into the significant potential of AI in the advancement of personalized medicine by effectively analyzing extensive sets of patient data, encompassing genomic and clinical information. The AI systems gather patient-specific data to offer personalized healthcare recommendations, thereby assisting in clinical decision-making and improving treatment precision [24]. To identify potential hit compounds against malignant glioma, a group of scientists employed a combination of molecular modeling methods and machine learning algorithms. They developed deep learning based QSAR models for identification of anti-cancer molecules against glioma [25]. DRIAD (drug repurposing in Alzheimer's disease) was introduced by Rodriguez *et al*. This machine learning framework evaluates the possibility of associations between the severity of AD pathology and molecular mechanisms in lists of gene names [26].

7. AI in Pharmaceutical Biotechnology

7.1. Relevant Studies

Sumitomo Dainippon Pharma collaborated with Exscientia to discover DSP-1181 for obsessive compulsive disorder in less than a quarter of the time usually required for drug discovery processes (less than 12 months compared to four and a half years). Their goal is to advance the molecule into Phase I trials [27]. Manzano *et al.* described that AI algorithms can predict the immunogenicity of biologic candidates, allowing researchers to identify and modify potentially problematic sequences or structures. This improves the chances of successful clinical translation [28]. AI is also transforming the development of cell and gene therapies by enabling the identification of optimal targets, payload designs, and delivery vehicles. The integration of AI is expected to significantly accelerate the advancement of these personalized treatment modalities [29]. Recent bioinformatics analyses have illuminated key genes and pathways that contribute to T-DM1 resistance in OE-19 esophageal cancer cells, underscoring the complexity of therapeutic resistance mechanisms [30]. A study has identified nearly 270 companies operating in the AI-driven drug discovery sector, with over half of these companies located in the United States. Additionally, significant hubs are emerging in Western Europe and Southeast Asia, highlighting the global expansion of this innovative industry [31]. AstraZeneca and BenevolentAI have been collaborating for a long time, and together they have identified several new targets in idiopathic pulmonary fibrosis. This collaboration has since expanded to cover other therapeutic areas as well [32]. In the context of COVID-19, matrix factorization-based feature selection methods have been deployed to decode the clinical biomarker space, providing insights into potential diagnostic and prognostic markers [33]. Tanoli *et al.* focus on the application of machine learning and AI methods, which depend on publicly accessible databases and information resources. This study pointed out the use of comprehensive target activity profiles to identify potent off-targets with therapeutic potential in drug repurposing [34].

8. AI in Pharmaceutical Nanotechnology

8.1. Relevant Studies

Researchers at Cardiff University, in collaboration with AstraZeneca, used artificial intelligence to design bespoke nanoparticles for delivering drug molecules, specifically mRNA, to cancer cells [35, 36]. This AI-designed nanoparticle proved to be more effective as a delivery shuttle compared to other prototypes. Yazdipour *et al.* analyzed 26 studies employing AI tools, primarily Random Forest and Support Vector Machine, to predict nanoparticle toxicity, focusing mainly on metal oxide and metallic nanoparticles. The AI models generally demonstrated acceptable performance, offering a robust, fast, and cost-effective alternative to traditional experimental methods for toxicity evaluation. The study highlights the potential of AI to streamline safety assessments in nanotechnology, addressing the need for more efficient toxicity prediction methods in this rapidly evolving field [37]. A number of studies have been conducted to explore the potential applications of AI in drug discovery. They underscore how pharmaceutical scientists, computer scientists, statisticians, physicians, and other experts are teaming up to investigate the potential of AI-based technologies for accelerating the drug development process and discovering new treatments [38, 39].

9. AI in Pharmacognosy

9.1. Relevant Studies

Desai et al. in 2022 discussed various AI mechanisms to enhance pharmacognostic performance, demonstrating AI's role in identifying active compounds and optimizing extraction processes [40]. Ferdous *et al.* created MproPred, a web application that uses machine learning to predict compound bioactivity against SARS-CoV-2's main protease. The tool, utilizing random forest algorithms, achieved robust predictive performance with 758 compounds. Additionally, the app screened the CMNPD marine compound database, revealing significant correlations between predicted bioactivity and binding affinity to Mpro, highlighting its potential for COVID-19 drug development [41].

10. AI in Pharmaceutical Management and Economics

10.1. Relevant Studies

Today, many pharmaceutical companies utilize AI tools to improve their manufacturing procedures. These tools can predict the ideal conditions to produce the drugs, monitor the drugs quality, and predict the potential issues before occurrence, which helps minimize waste and enhance the overall efficiency of the process. Computer control, process analytical technology (PAT), and algorithms play a vital role in developing automated and integrated manufacturing platforms for pharmaceuticals. This not only speeds up the manufacturing process but also enhances the understanding of reaction mechanisms while saving costs, materials, and manpower [42, 43]. A study on the many-objective optimization of a three-echelon pharmaceutical supply chain showed significant improvements in efficiency and cost reduction. The AI-driven approach optimized inventory levels and streamlined distribution processes [44]. Guo in 2023 explored AI's value in optimizing pharmaceutical supply chains, demonstrating reductions in lead times and inventory costs. Case studies within the paper illustrated practical benefits and implementation strategies [45]. Kumar *et al.* in 2022 examined AI's role in managing healthcare supply chains, including a case study of the US pharmaceutical supply chain post-Hurricane Maria. AI tools significantly improved supply chain responsiveness and reliability [46].

References

1. Harren T, Matter H, Hessler G, Rarey M, Grebner C: **Interpretation of Structure-Activity Relationships in Real-World Drug Design Data Sets Using Explainable Artificial Intelligence**. *J Chem Inf Model* 2022, **62**(3):447-462.
2. Matsuzaka Y, Uesawa Y: **A Deep Learning-Based Quantitative Structure-Activity Relationship System Construct Prediction Model of Agonist and Antagonist with High Performance**. *Int J Mol Sci* 2022, **23**(4).
3. Griffin DJ, Coley CW, Frank SA, Hawkins JM, Jensen KF: **Opportunities for Machine Learning and Artificial Intelligence to Advance Synthetic Drug Substance Process Development**. *Organic Process Research & Development* 2023, **27**(11):1868-1879.
4. Thakkar A, Johansson S, Jorner K, Buttar D, Reymond J-L, Engkvist O: **Artificial intelligence and automation in computer aided synthesis planning**. *Reaction chemistry & engineering* 2021, **6**(1):27-51.
5. Johansson S, Thakkar A, Kogej T, Bjerrum E, Genheden S, Bastys T, Kannas C, Schliep A, Chen H, Engkvist O: **AI-assisted synthesis prediction**. *Drug Discovery Today: Technologies* 2019, **32-33**:65-72.

6. Wang S, Wang L, Li F, Bai F: **DeepSA: a deep-learning driven predictor of compound synthesis accessibility**. *Journal of Cheminformatics* 2023, **15**(1):103.
7. Lee N, Jeong J, Shin D: **AI System for Supporting Generation of Optimal Synthetic Pathways Based on Chemical Reaction Big Data**. In: *Computer Aided Chemical Engineering.* Edited by Türkay M, Gani R, vol. 50: Elsevier; 2021: 1053-1058.
8. Tsou LK, Yeh S-H, Ueng S-H, Chang C-P, Song J-S, Wu M-H, Chang H-F, Chen S-R, Shih C, Chen C-T, Ke Y-Y: **Comparative study between deep learning and QSAR classifications for TNBC inhibitors and novel GPCR agonist discovery**. *Scientific Reports* 2020, **10**(1):16771.
9. Mao J, Akhtar J, Zhang X, Sun L, Guan S, Li X, Chen G, Liu J, Jeon H-N, Kim MS *et al*: **Comprehensive strategies of machine-learning-based quantitative structure-activity relationship models**. *iScience* 2021, **24**(9):103052.
10. Selvaraj C, Elakkiya E, Prabhu P, Velmurugan D, Singh SK: **Chapter 9 - Advances in QSAR through artificial intelligence and machine learning methods**. In: *QSAR in Safety Evaluation and Risk Assessment.* Edited by Hong H: Academic Press; 2023: 101-116.
11. Niazi SK, Mariam Z: **Recent Advances in Machine-Learning-Based Chemoinformatics: A Comprehensive Review**. *Int J Mol Sci* 2023, **24**(14).
12. Dong J, Wu Z, Xu H, Ouyang D: **FormulationAI: a novel web-based platform for drug formulation design driven by artificial intelligence**. *Briefings in Bioinformatics* 2023, **25**(1).
13. Reddy K, Harichandana U, Alekhya T, Rajesh S: **A study of robotic process automation among artificial intelligence**. *International Journal of Scientific and Research Publications* 2019, **9**(2):392-397.
14. Goud JS, Elisetti SK, Kaur N, Singh R, Saini KS, Arora V: **Artificial Intelligence in Formulation and Product Design of BCS Class I and II Drugs**. In: *Micro-Electronics and Telecommunication Engineering: 2023// 2023; Singapore*. Springer Nature Singapore: 481-494.
15. Khoshdel F, Mottaghi-Dastjerdi N, Yazdani F, Salehi S, Ghorbani A, Montazeri H, Soltany-Rezaee-Rad M, Goodarzy B: **CTGF, FN1, IL-6, THBS1, and WISP1 genes and PI3K-Akt signaling pathway as prognostic and therapeutic targets in gastric cancer identified by gene network modeling**. 2023.
16. Amerizadeh F, Rahmani F, Ghorbani A, Mottaghi-Dastjerdi N, Bazyari MJ, Rouhani J, Pasdar A: **A Prospective to Regulatory Role of Mirnas on Wnt/B-Catenin Signaling and its Crosstalk to the Other Cellular Pathways in Tumorigenesis of Glioblastoma by a Systems Biology Approach**. *B-Catenin Signaling and its Crosstalk to the Other Cellular Pathways in Tumorigenesis of Glioblastoma by a Systems Biology Approach*.
17. de Jong J, Cutcutache I, Page M, Elmoufti S, Dilley C, Fröhlich H, Armstrong M: **Towards realizing the vision of precision medicine: AI based prediction of clinical drug response**. *Brain* 2021, **144**(6):1738-1750.
18. Born J, Markert G, Janakarajan N, Kimber TB, Volkamer A, Martínez MR, Manica M: **Chemical representation learning for toxicity prediction**. *Digital Discovery* 2023, **2**(3):674-691.
19. Liu M, Wu Y, Chen Y, Sun J, Zhao Z, Chen X-w, Matheny ME, Xu H: **Large-scale prediction of adverse drug reactions using chemical, biological, and phenotypic properties of drugs**. *Journal of the American Medical Informatics Association* 2012, **19**(e1):e28-e35.
20. Vijayan RSK, Kihlberg J, Cross JB, Poongavanam V: **Enhancing preclinical drug discovery with artificial intelligence**. *Drug Discovery Today* 2022, **27**(4):967-984.
21. Kumar A, Kini SG, Rathi E: **A Recent Appraisal of Artificial Intelligence and In Silico ADMET Prediction in the Early Stages of Drug Discovery**. *Mini Rev Med Chem* 2021, **21**(18):2788-2800.
22. Yin J, Ngiam KY, Teo HH: **Role of Artificial Intelligence Applications in Real-Life Clinical Practice: Systematic Review**. *J Med Internet Res* 2021, **23**(4):e25759.
23. Shapiro MA, Stuhlmiller TJ, Wasserman A, Kramer GA, Federowicz B, Hoos W, Kaufman Z, Chuyka D, Mahoney W, Newton ME: **AI-augmented clinical decision support in a patient-centric precision oncology registry**. *AI in Precision Oncology* 2024, **1**(1):58-68.

24. Parekh A-DE, Shaikh OA, Simran, Manan S, Hasibuzzaman MA: **Artificial intelligence (AI) in personalized medicine: AI-generated personalized therapy regimens based on genetic and medical history: short communication**. *Annals of Medicine and Surgery* 2023, **85**(11):5831-5833.
25. Zaka M, Mohammed F, Fareed AF, Durdagi S: **Development of deep learning based QSAR models for the identification of novel anti-cancer therapeutics against malignant glioma from ultra large libraries**. *Biophysical Journal* 2022, **121**(3):529a.
26. Rodriguez S, Hug C, Todorov P, Moret N, Boswell SA, Evans K, Zhou G, Johnson NT, Hyman BT, Sorger PK *et al*: **Machine learning identifies candidates for drug repurposing in Alzheimer's disease**. *Nature Communications* 2021, **12**(1):1033.
27. Exscientia: **Sumitomo Dainippon Pharma and Exscientia joint development new drug candidate created using artificial intelligence (AI) begins clinical trial**. In.
28. **Artificial Intelligence in the Biopharmaceutical Industry: Treacherous or Transformative?** [https://www.bioprocessintl.com/information-technology/artificial-intelligence-in-the-biopharmaceutical-industry-treacherous-or-transformative-]
29. Herbrand U: **What's Hot in 2024: Artificial Intelligence for ATMPs**. *Eureka* 2023.
30. Yazdani F, Mottaghi-Dastjerdi N, Ghorbani A, Soltany-Rezaee-Rad M, Montazeri H, Khoshdel F, Guzzi PH: **Identification of Key Genes and Pathways Governing T-DM1 Resistance in OE-19 Esophageal Cancer Cells through Bioinformatics Analysis**. 2023.
31. Alex Devereson EI, Matej Macak, Navraj Nagra, and Erika Stanz: **AI in biopharma research: A time to focus and scale**. *MacKinesey & Company, Life Sciences* 2022.
32. BenevolentAI: **BenevolentAI announces 3-year collaboration expansion with AstraZeneca focused on systemic lupus erythematosus and heart failure**. In.; 2022.
33. Saberi-Movahed F, Mohammadifard M, Mehrpooya A, Rezaei-Ravari M, Berahmand K, Rostami M, Karami S, Najafzadeh M, Hajinezhad D, Jamshidi M: **Decoding clinical biomarker space of COVID-19: Exploring matrix factorization-based feature selection methods**. *Computers in biology and medicine* 2022, **146**:105426.
34. Tanoli Z, Vähä-Koskela M, Aittokallio T: **Artificial intelligence, machine learning, and drug repurposing in cancer**. *Expert Opin Drug Discov* 2021, **16**(9):977-989.
35. **Delivering the next wave of scientific innovation: IMED Annual Review 2017** [https://www.astrazeneca.com/content/dam/az/PDF/2017/IMED_Annual%20Review%202017_FINAL%20APPROVED.pdf]
36. **AI-generated nanoparticles prove capable of delivering modern medicines to diseased cells** [https://www.cardiff.ac.uk/news/view/2728830-ai-generated-nanoparticles-prove-capable-of-delivering-modern-medicines-to-diseased-cells]
37. Banaye Yazdipour A, Masoorian H, Ahmadi M, Mohammadzadeh N, Ayyoubzadeh SM: **Predicting the toxicity of nanoparticles using artificial intelligence tools: a systematic review**. *Nanotoxicology* 2023, **17**(1):62-77.
38. Farghali H, Kutinová Canová N, Arora M: **The potential applications of artificial intelligence in drug discovery and development**. *Physiol Res* 2021, **70**(Suppl4):S715-s722.
39. Paul D, Sanap G, Shenoy S, Kalyane D, Kalia K, Tekade RK: **Artificial intelligence in drug discovery and development**. *Drug Discov Today* 2021, **26**(1):80-93.
40. Desai N, Patel D, Vyas N: **Pharmacognosy with Artificial Intelligence: An Interdisciplinary Approach**. In: *Advances in Data and Information Sciences: Proceedings of ICDIS 2021*. Springer; 2022: 167-177.
41. Ferdous N, Reza MN, Hossain MU, Mahmud S, Napis S, Chowdhury K, Mohiuddin A: **Mpropred: A machine learning (ML) driven Web-App for bioactivity prediction of SARS-CoV-2 main protease (Mpro) antagonists**. *Plos one* 2023, **18**(6):e0287179.
42. Liu P, Jin H, Chen Y, Wang D, Yan H, Wu M, Zhao F, Zhu W: **Process analytical technologies and self-optimization algorithms in automated pharmaceutical continuous manufacturing**. *Chinese Chemical Letters* 2024, **35**(3):108877.

43. Kehayov M, Holder L, Koch V: **Application of artificial intelligence technology in the manufacturing process and purchasing and supply management**. *Procedia Computer Science* 2022, **200**:1209-1217.
44. Santos JAM, Sousa JMC, Vieira SM, Ferreira AF: **Many-objective optimization of a three-echelon supply chain: A case study in the pharmaceutical industry**. *Computers & Industrial Engineering* 2022, **173**:108729.
45. Guo W: **Exploring the Value of AI Technology in Optimizing and Implementing Supply Chain Data for Pharmaceutical Companies**. *Innovation in Science and Technology* 2023, **2**(3):1-6.
46. Kumar A, Mani V, Jain V, Gupta H, Venkatesh VG: **Managing healthcare supply chain through artificial intelligence (AI): A study of critical success factors**. *Comput Ind Eng* 2023, **175**:108815.
